# Knowledge level and access barriers related to sexual and reproductive health information among youth with disabilities in China: a cross-sectional study

**DOI:** 10.1186/s12978-023-01625-9

**Published:** 2023-06-06

**Authors:** Wenjuan Qi, Hongyan Li, Qiguo Lian, Xiayun Zuo, Chunyan Yu, Chaohua Lou, Xiaowen Tu

**Affiliations:** 1 NHC Key Laboratory of Reproduction Regulation (Shanghai Institute for Biomedical and Pharmaceutical Technologies), Fudan University, 779 Old Hu Min Road, Shanghai, 200237 China; 2 Shanghai Municipal Center for Disease Control & Prevention, 1380 West Zhongshan Road, Shanghai, 200336 China; 3 UNESCO Beijing Office, 5-153 Jianguomenwai DRC, Beijing, 100600 China

**Keywords:** Disability, Sexual and reproductive health, Knowledge, Sexuality education, Barriers, Youth, China

## Abstract

**Background:**

Youth with disabilities have the same right to sexual and reproductive health (SRH) as their peers without disabilities. However, their needs and rights are often ignored. Little is known about the knowledge, needs and access barriers related to SRH information among youth with different types of disabilities in China.

**Methods:**

A cross-sectional survey was carried out among 473 unmarried youth aged 15–24 with visual, hearing, physical disabilities in both urban and rural areas in China.

**Results:**

Out of a maximum possible score of 100, respondent’s median score ranged from 30 to 50 for knowledge related to sexual physiology, STIs/HIV/AIDS and contraception. For these three categories of knowledge, respondents with hearing and physical disabilities or from rural areas scored lower than their counterparts with visual disabilities or from urban areas. The multivariate analyses showed that the residential area and education level were strong correlates of knowledge among respondents with visual and hearing disabilities. Other significant correlates were age for respondents with visual impairment and physical impairment, and single child status in the family and father’s education level for respondents with hearing impairment. Sources of and barriers and preferences in accessing SRH information differed by type of disabilities, residential areas and gender. In general, school teachers were the primary and most preferred sources of SRH knowledge, followed by the Internet, peers/friends and parents. Unaware of where to get accurate information and feeling embarrassed to seek information were the two most frequently mentioned barriers in accessing SRH information.

**Conclusion:**

Respondents had poor knowledge of SRH and limited access to SRH information, especially those from rural areas. Efforts should be made to promote school and family-based sexuality education tailored for youth with different types of disabilities.

## Plain language summary

Youth with disabilities have the same sexual and reproductive health (SRH) needs as their peers without disabilities and the equal right to attain the highest standard of SRH. However, their SRH needs and rights are often overlooked or neglected. Studies on SRH among unmarried youth with disabilities are very limited in China. This study used data collected from 473 unmarried youths with different types of disabilities from both urban and rural areas in China, to understand their SRH knowledge and its associated factors, as well as barriers and preferences in accessing sexuality-related information. This study demonstrated that unmarried youth with disabilities lacked knowledge of SRH, especially those with hearing or physical disabilities and those from rural areas. In general, residential area and education level were significant correlates of knowledge among respondents. Sources of and barriers and preferences in accessing SRH information varied across types of disabilities, residential areas and gender. In general, school teachers were the primary and most preferred sources of knowledge, followed by the Internet, peers and parents. Unaware of the available sources of accurate information and feeling embarrassed to seek information were the most frequently mentioned barriers in accessing SRH information. The findings of the study highlight the need to reduce the barriers to sexuality education for unmarried youth with different types of disabilities. Efforts should be made to promote school and family-based sexuality education tailored for youth with different types of disabilities and empower them to seek information proactively.

## Background

The Programme of Action (PoA) of the International Conference on Population and Development (ICPD), held in Cairo in 1994, explicitly called for governments at all levels to consider the needs and rights of all people in achieving sustainable development and affirmed that sexual and reproductive health (SRH) and rights are fundamental to people’s survival, health and wellbeing. The PoA also gave high priority to adolescents' SRH and rights and drew attention to the elimination of specific forms of discrimination that persons with disabilities may face regarding reproductive health and rights [1]. One hundred and seventy-nine governments and civil society partners including the Chinese government made the commitment to “sexual and reproductive health for all by the year 2015” at the conference. The commitment to the PoA by the governments and civil society partners was further reinforced at the 25th anniversary of the ICPD in 2019. Specific objectives were also captured under Sustainable Development Goal 3 and 5 of the 2030 Agenda [2]. The Convention on the Rights of Persons with Disabilities (CRPD), adopted by the United Nations General Assembly in December 2006, also underscored the need of ensuring SRH and rights of persons with disabilities [3]. China was one of the first countries to sign the CRPD in 2007 and ratify it in 2008.

Persons with disabilities have the same SRH needs as other people and equal rights to attain the highest standard of SRH. Despite the above-mentioned commitment, persons with disabilities face more barriers than their peers without disabilities in accessing SRH information and services, mainly due to discrimination and misunderstanding by the people around them rather than their disabilities [4–6]. For example, persons with disabilities are often assumed to be asexual or lacking sexual ability and therefore do not need SRH services [6–9]. The belief and the consequent attitudes lead to a series of adverse outcomes for people with disabilities, such as people’s reluctance to build intimate relationships with people with disabilities, and exclusion of persons with disabilities from SRH programs. As the most marginalized subgroups of persons with disabilities, adolescents and unmarried young adults are the most likely excluded groups [4, 10, 11]. Studies show that young people with disabilities have the same or higher rate of sexual intercourse compared with their peers without disabilities [5, 12–14]. Furthermore, they are more likely to experience sexual abuse and other risks [4, 12, 15–17] and need to learn how to deal with reduced privacy due to disabilities [18]. Therefore, they may need SRH education and services more than their peers without disabilities. But the SRH needs and rights of persons with disabilities are often overlooked or neglected. Young people with disabilities tend to receive sexuality education at a lesser degree than their peers without disabilities even in European countries with a longer history of sexuality education [6, 19–21]. Compared with the general population, they lack SRH knowledge and are more vulnerable to the negative consequences of sexual behavior such as sexually transmitted infections (STIs) and unwanted pregnancies [9, 22]. To promote sexuality education for young people with disabilities, evidence is required on the barriers they face in accessing SRH information. Barriers to sexuality education for young people with disabilities vary by country, culture and disability types. Unfortunately, most of the studies on barriers to sexuality education for young people with disabilities were conducted in the US and European countries, and the research focus was mainly on intellectual and developmental disabilities and autism spectrum disorders with a gap in the research of other type of disabilities [6, 23].

China has a population of 85.64 million living with disabilities [24, 25]. As a country deeply influenced by the Confucian culture, sex remains a taboo topic. Sexuality education was not officially introduced into schools until 1980s in China. In 1988, the State Education Commission and the State Family Planning Commission jointly promulgated the Notice on Puberty Health Education in Secondary Schools, mandating three topics in terms of the educational content: sexual biology, psychology, and ethics [26]. Many laws and regulations adopted or revised after ICPD in 1994 included articles on the health and development rights of adolescents [26, 27]. The Health Education Guidelines for Primary and Secondary Schools issued by the Ministry of Education in 2008 stipulate that puberty health shall be a major component of primary and secondary school health education [26]. Despite this progress, the content of school sexuality education was not comprehensive, with insufficient coverage of topics such as gender, sexual orientation, violence, rights, sexual behavior, contraceptive methods, pregnancy and abortion. In the meantime, school-based sexuality education was not always guaranteed because of the myths of sexuality education, culture barrier, lack of textbooks or curriculum standards and trained teachers [26]. The SRH needs and rights of young people with disabilities have been neglected by both the disability community and those working on SRH. Although a significant amount of data is available on adolescents’ SRH, there is a lack of data in China on SRH among young people with disabilities. The sensitivity regarding sexuality of young people and disability makes it more challenging to conduct such research. The objective of this study is to understand the SRH knowledge and its associated factors, as well as barriers and preferences in accessing sexuality-related information among unmarried youth with different types of disabilities in both urban and rural areas in China, so as to provide evidence for promoting sexuality education that takes disability into full considerations.

## Methods

### Study design and respondents

The cross-sectional survey was conducted among unmarried young people aged 12–24 with visual, hearing and physical disabilities. Considering this was the first study in China in terms of sexuality and disability, focus group discussions and in-depth interviews with young people with disabilities and in-depth interviews with their teachers and parents were conducted to inform the design of the quantitative survey. The results of qualitative interviews were published elsewhere [28–30]. All eligible respondents held disability cards issued by the China Disabled Persons’ Federation, which certified their levels of disability. The study excluded people with multiple disabilities.

Respondents of the survey were recruited from both urban areas (Shanghai Municipality) and rural areas (rural areas of Xianyang, Weinan and Baoji cities in Shaanxi province) in China. These sites were selected in consideration of the level of support from local Disabled Persons’ Organizations (DPOs), communities and schools. In China, young people with disabilities are either enrolled in regular schools, special schools and rehabilitation training institutions, working in welfare-oriented or disability-friendly enterprises, or staying at home, depending on their level of disability, the local availability of special schools, and welfare-oriented or disability-friendly enterprises. Given the challenges of conducting studies on sexuality among unmarried young people with disabilities, convenient sampling was used to recruit participants. Participants with visual and hearing disabilities were recruited from five special education schools including three from urban and two from rural areas, while respondents with physical disabilities were recruited from two special education schools, two communities and one sports training center for the disabled in urban areas, and welfare-oriented or disability-friendly enterprises and regular schools and communities in rural areas. All eligible respondents in the selected schools, enterprises and communities were recruited for the study. A total of 699 respondents participated in the survey, with 126 of them aged 12–14 years and 473 aged 15–24 years. This paper is based on the analysis of the data concerning the sub-sample of 473 respondents aged 15–24 years, including 158 youth with visual impairment, 207 with hearing impairment and 108 with physical disability.

### Procedure

The study used an anonymous structured questionnaire to collect data. The questionnaire was initially designed by two age groups (12–14 and 15–24), and was revised and finalized following a face validity study and a pilot test involving male and female young people with different types of disability of both age groups. The survey was administered in an accessible way to the respondents and the data were collected from May to June 2015. For respondents with visual impairment, those with low vision filled a self-administrated paper questionnaire in big fonts and those who were blind filled a self-administrated electronic questionnaire via computer software or an interviewer-administered questionnaire by listening to tape recorders. The respondents with hearing impairment answered a self-administered paper questionnaire with the help of interpreters and interviewers. Most of the respondents with physical disability answered a self-administrated paper questionnaire while those with difficulties in completing the questionnaire on their own answered an interviewer-administered questionnaire. Trained investigators were present to assist participants who had difficulties with filling out the questionnaire and to check and ensure the completion of the questionnaires. This study was reviewed and approved by the institutional review board of Shanghai Institute of Planned Parenthood Research (PJ2014–04). Informed consent or assent was obtained from the participants, parents as well as school teachers before data collection.

### Measurements

*Socio-demographic characteristics* Respondents were asked about their gender, date of birth, residential area, student status, education level, disability onset date, single-child status, cohabitants, parents’ education level and disability status.

*SRH knowledge* Respondents were asked three categories of SRH knowledge—sexual physiology (menstruation, nocturnal emissions, masturbation, pregnancy and childbirth), sexually transmitted infections (STIs)/HIV/AIDS (transmission modes and prevention methods) and contraception (contraceptive methods and where to get contraceptives). The three categories of knowledge were assessed by 9, 16 and 14 single-choice questions respectively. For each question, 1 point was awarded to the correct answer and 0 point to the incorrect or “do not know” answer. The sum of the points for each category or all three categories was then converted into a scaled score ranging from 0 to 100 (100 is the highest possible score), with higher scores indicating higher levels of knowledge.

*Sources of, barriers and preference in accessing SRH Knowledge* Respondents were asked about their main sources of SRH knowledge, barriers in accessing sexuality-related information, and their preferred sources, contents and ways when acquiring sexuality-related information.

### Statistical analysis

The SAS 9.3 (SAS Institute Inc., Cary, NC, USA) was used for statistical analysis. The data was analyzed by type of disability. Subgroup differences were analyzed using χ^2^-test, Wilcoxon rank sum test and Kruskal–Wallis H rank sum test as appropriate. Multivariate ordinal logistic regression model was used to examine the association between socio-demographic variables and SRH knowledge level using quartiles or tertiles as the cutoff points to divide the data set into four or three equal groups depending on the test of the proportional odds assumption. For each variable included in the regression models, odds ratio (OR) and its 95% confidence interval (95% CI) were calculated. All analyses employed an alpha level for statistical significance of 0.05 (two-tailed).

## Results

### Socio-demographic characteristics of the respondents

Around half of the respondents with hearing and physical disabilities were male, but more than 70% of the respondents with visual impairment were male. More than half of the respondents were from urban areas, with the percentage for respondents with hearing impairment (61.4%) significantly higher than that for the visually impaired (41.8%). The mean age of respondents was 18.89 (± 2.41) years old with more than half of them aged 15–19 years. All respondents with visual and hearing impairment were students and most of them had an education level of senior high school or above, while the corresponding percentage among the respondents with physical disability was only around fifty percent. Less than half (46.5%) of all respondents were the only child in the family with the percentage of the visually impaired (39.2%) and physically disabled (38.9%) much lower than that of the hearing impaired (56.0%). More than two-thirds of the respondents were living with parents and more than 60% of their parents had an education level of junior high school or above. Nearly half of the respondents were born with a disability, with the percentage of the visually impaired (61.4%) significantly higher than that of the hearing impaired (41.1%) and physically disabled (44.4%). More than 70% of the respondents reported their parents were without disabilities (Table 1).

**Table 1:** Characteristics of study respondents, by type of disabilities (n, %)

Characteristics	Visual (n = 158)	Hearing (n = 207)	Physical (n = 108)	Total (n = 473)
*Gender*				
Male	113 (71.5)	101 (48.8)	54 (50.0)^***^	268 (56.7)
Female	45 (28.5)	106 (51.2)	54 (50.0)	205 (43.3)
*Age group*				
15 ~ 19	99 (62.7)	104 (50.2)	60 (55.6)	263 (55.6)
20 ~ 24	59 (37.3)	103 (49.8)	48 (44.4)	210 (44.0)
*Residence*				
Urban	66 (41.8)	127 (61.4)	60 (55.6)	253 (53.5)
Rural	92 (58.2)	80 (38.6)	48 (44.4)	220 (46.5)
*Student*				
Yes	158(100.0)	207(100.0)	53(49.1)	418(88.4)
No	0(0.0)	0(0.0)	55(50.9)	55(11.6)
*Single child*				
Yes	62 (39.2)	116 (56.0)	42 (38.9)	220 (46.5)
No	96 (60.8)	91 (44.0)	66 (61.1)	253 (53.5)
*Education*				
Junior high or lower	24 (15.2)	63(30.4)	51 (47.2)^***^	140 (29.6)
Senior high or above	134 (84.8)	144 (69.6)	57 (52.8)	333 (70.4)
*Living circumstance*				
With parents	102 (64.6)	145 (70.0)	75 (69.4)^***^	322 (68.1)
With grandparents	6 (3.8)	15 (7.2)	9 (8.3)	30 (6.3)
With classmates	45 (28.5)	27 (13.0)	6 (5.6)	78(16.5)
Alone	2 (1.3)	12 (5.8)	8 (7.4)	22 (4.7)
With others	3 (1.9)	8 (3.9)	10 (9.3)	21 (4.4)
*Disability onset*				
From birth	97 (61.4)	85 (41.1)	48 (44.4)^***^	230 (48.6)
< 10 years	27 (17.1)	69 (33.3)	23 (21.3)	119 (25.2)
≥ 10 years	28 (17.7)	7 (3.4)	17 (15.7)	52 (11.0)
Unknown	6 (3.8)	46 (22.2)	20 (18.5)	72 (15.2)
*Father’s education*				
Primary or lower	27 (17.1)	26 (12.6)	16 (14.8) ^*^	69 (14.6)
Junior high	51 (32.3)	69 (33.3)	34 (31.5)	154 (32.6)
Senior high	39 (24.7)	51 (24.6)	27 (25.0)	117 (24.7)
College or above	27(17.1)	15(7.2)	10 (9.3)	52(11.0)
Unknown	14 (8.9)	46 (22.2)	21 (19.4)	81 (17.1)
*Mother’s education*				
Primary or lower	34 (21.5)	45 (21.7)	23 (21.3)^**^	102 (21.6)
Junior high	40 (25.3)	71 (34.3)	34 (31.5)	145 (30.7)
Senior high	46 (29.1)	29 (14.0)	23 (21.3)	98 (20.7)
College or above	21(13.3)	17(8.2)	9 (8.3)	47 (9.9)
Unknown	17 (10.8)	45 (21.7)	19 (17.6)	81 (17.1)
*Parents’ disability*				
One or both parents	31 (19.6)	70 (33.8)	34 (31.5)	135 (28.5)
None	127 (80.4)	137 (66.2)	74 (68.5)	338 (71.5)

*p < 0.05; **p < 0.01; ***p < 0.001

### Knowledge on SRH

Out of a maximum possible score of 100, the median scores of respondents ranged from 30 to 50 for knowledge related to sexual physiology, STIs/HIV and contraception, with STIs/HIV/AIDS registering the highest score. Among respondents with different types of disabilities, visually impaired respondents had the highest scores for all three categories of knowledge, and the respondents with hearing impairment scored higher for contraceptive knowledge than those with physical disabilities. Respondents in urban areas scored higher for each category of knowledge than their rural counterparts. Gender difference was not observed (Table 2).

**Table 2:** SRH knowledge of youth with disabilities [M(Q_L_,Q_U_)]

Knowledge	Type of disabilities			Residence		Gender		Total
	Visual	Hearing	Physical	Urban	Rural	Male	Female	
Sexual physiology	44.4 (22.2, 66.7)^a^	33.3 (11.1, 55.6)^b^	33.3 (11.1, 55.6)^b***^	44.4 (22.2, 66.7)	33.3 (11.1, 44.4)^***^	33.3 (11.1, 55.6)	33.3 (11.1, 55.6)	33.3 (11.1, 55.6)
STIs/HIV/AIDS	60.0 (40.0, 70.0)^a^	40.0 (0.0, 60.0)^b^	40.0 (0.0, 65.0)^b***^	60.0 (40.0, 70.0)	40.0 (0.0, 60.0)^***^	50.0 (30.0, 70.0)	50.0 (10.0, 70.0)	50.0 (20.0, 70.0)
Contraception	42.9 (21.4, 64.3)^a^	35.7 (14.3, 50.0)^b^	28.6 (10.7, 50.0)^c***^	42.9 (21.4, 64.3)	28.6 (14.3, 42.9)^***^	35.7 (14.3, 57.1)	35.7 (14.3, 57.1)	35.7 (14.3, 57.1)
Total	48.5 (33.3, 63.6)^a^	36.4 (18.2, 51.5)^b^	36.4 (9.1, 48.5)^b***^	48.5 (33.3, 63.6)	33.3 (15.1, 45.4)^***^	39.4 (21.2, 54.5)	39.4 (21.2, 57.6)	39.4 (21.2, 57.6)

There are significant differences between groups with different letters of a, b or c; ^***^ p < 0.001

### Sources of, barriers and preference in accessing SRH information

Sources of and barriers and preferences in accessing SRH information differed by type of disabilities (Table 3). School teachers were the most frequently reported to be the primary source of SRH information by all respondents regardless of their types of disability, followed by the Internet, parents and books/magazines. Respondents with physical disabilities were significantly less likely to get SRH information from peers than other respondents. The most frequently mentioned barriers in accessing SRH information, across disability types, were not knowing where to get accurate information and feeling embarrassed to seek information. The respondents with hearing impairment were significantly more likely to face these two barriers than their counterparts with visual and physical disabilities. In general, respondents with visual impairment had higher needs for SRH information than their counterparts with hearing impairment or physical disabilities. The top 4 SRH topics that all the respondents would like to acquire information about were puberty change and health care, friendship/love/marriage, sexual harassment/abuse/self-protection and STIs/HIV/AIDS prevention. The top four preferred sources of SRH information were teachers, the Internet, peers/classmates/friends and parents among all respondents, but more respondents with visual impairment preferred the Internet, while more respondents with hearing impairment preferred teachers and peers/classmates/friends, and more respondents with physical disability preferred parents as sources of information. With regards to the top four preferred ways of acquiring SRH information, class/lecture, books/newspaper, TV/video and the Internet were frequently mentioned by all respondents. While broadcast/audio was frequently reported by respondents with visual impairment, discussion/activity was frequently reported by respondents with hearing and physical disability as preferred ways of obtaining SRH information.

**Table 3:** Sources of, difficulties and preferences in accessing SRH information for youth with disabilities (n, %)

	Type of disabilities			Residence		Gender		Total
	Visual	Hearing	Physical	Urban	Rural	Male	Female	
*Main sources*								
Teachers	95 (60.1)	126 (60.0)	56 (51.9)	186(73.5)	91(41.4)^***^	143(53.4)	134 (65.4)^**^	277 (58.6)
Peers/classmates/friends	55 (34.8)	84 (40.6)	22 (20.4)^**^	96(37.9)	65(29.5)	92(34.3)	69(33.7)	161 (34.0)
Internet	58 (36.7)	64 (30.9)	38 (35.2)	77(30.4)	83(37.7)	103(38.4)	57(27.8)^*^	160 (33.8)
Parents	51 (32.3)	66 (31.9)	30 (27.8)	89(35.2)	58(26.4)^*^	61(22.8)	86(42.0)^***^	147 (31.1)
Books/magazines	47 (29.7)	54 (26.1)	26 (24.1)	74(29.2)	53(24.1)	70(26.1)	57(27.8)	127 (26.8)
TV/ broadcast/radio	39 (24.7)	37 (17.9)	28 (25.9)	52(20.6)	52(23.6)	67(25.0)	37(18.0)	104 (22.0)
Professionals/hotline	22 (13.9)	30 (14.5)	12 (11.1)	33(13.0)	31(14.1)	34(12.7)	30(14.6)	64 (13.5)
Siblings/relatives	6 (3.8)	22 (10.6)	8 (7.4)	19(7.5)	17(7.7)	19(7.1)	17(8.3)	36 (7.6)
Posters /exhibitions	4 (2.5)	17 (8.2)	5 (4.6)	20(7.9)	6(2.7)^*^	16(6.0)	10(4.9)	26 (5.5)
None of the above listed	5 (3.2)	17 (8.2)	11 (10.2)	9(3.6)	24(10.9)^**^	19(7.1)	14(6.8)	33 (7.0)
*Barriers*								
Unaware of the sources	51(32.3)	77 (37.2)	24 (22.2)^**^	62(24.5)	90(40.9)^***^	84(31.3)	68(33.2)	152 (32.1)
Feeling embarrassed	45 (28.5)	85 (41.1)	36 (33.3)^*^	79(31.2)	87(39.5)	75(28.0)	91(44.4)^***^	166 (35.1)
Getting little from school	29 (18.4)	57 (27.5)	21 (19.4)	68(26.9)	39(18.0)^*^	65(24.3)	42(20.5)	107 (22.6)
Hard to understand	24 (15.2)	45 (21.7)	18 (16.7)	45(17.8)	42(19.1)	49(18.3)	38(18.5)	87 (18.4)
Lack appropriate books	26 (16.5)	34 (16.4)	20 (18.5)	39(15.4)	41(18.6)	44(16.4)	36(17.6)	80 (16.9)
Conservative parents	18 (11.4)	31 (15.0)	14 (13.0)	38(15.0)	25(11.4)	29(10.8)	34(16.6)	63 (13.3)
*SRH information needs*								
Reproductive system	67(42.4)	58(28.4)	18(16.8)^***^	80(31.9)	63(28.9)	93(35.0)	50(24.6)^*^	143(30.5)
Puberty changes/health care	99(62.7)	136(66.7)	52(48.0)^**^	163(64.9)	124(56.9)	157(59.0)	130(64.4)	287(61.2)
Friendship/love/marriage	85(53.8)	80(39.2)	46(43.0)^*^	128(51.0)	83(38.1)^**^	125(47.0)	86(42.4)	211(45.0)
Pregnancy and childbirth	49(31.0)	53(26.0)	18(16.8)^*^	78(31.1)	42(19.3)^**^	58(21.8)	62(30.4)^*^	120(25.6)
Contraception and abortion	53(33.5)	66(32.4)	14(13.1)^***^	86(34.3)	47(21.6)^**^	62(23.3)	71(35.0)^**^	133(28.4)
STIs/HIV/AIDS prevention	68(43.0)	85(41.7)	27(25.2)^**^	99(39.4)	81(37.2)	94(35.3)	86(42.4)	180(38.4)
Sexual harassment/abuse/ self-protection	67(42.4)	100(49.0)	28(26.2)^***^	121(48.2)	74(33.9)^**^	86(32.3)	109(53.7)^***^	195(41.6)
Sexual orientation	34(21.5)	39(19.1)	12(11.2)	59(23.5)	26(11.9)^**^	35(13.2)	50(24.6)^**^	85(18.1)
*Preferred sources*								
Parents	43(27.2)	68(33.0)	45(41.7)^*^	93(36.9)	63(28.6)	71(26.6)	85(41.5)^***^	156(33.1)
School/teachers	86(54.4)	131(63.6)	53(49.1)^*^	163(64.7)	107(48.6)^***^	146(54.7)	124(60.5)	270(57.2)
Peers/classmates/friends	55(34.8)	86(41.7)	29(26.9)^*^	92(36.5)	78(35.5)	95(35.6)	75(36.6)	170(36.0)
Internet	91(57.6)	93(45.1)	46(42.6)^*^	112(44.4)	118(53.6)^*^	142(53.2)	88(42.9)^*^	230(48.7)
Community	15(9.5)	30(14.6)	9(8.3)	31(12.3)	23(10.5)	27(10.1)	27(13.2)	54(11.4)
Hospital	45(28.5)	59(28.6)	23(21.3)	77(30.6)	50(22.7)	55(20.6)	72(35.1)^***^	127(26.9)
*Preferred ways*								
Class/lecture	83(52.5)	117(56.8)	43(40.6)^*^	163(65.2)	80(36.4)^***^	120(44.9)	123(60.6)^***^	243(51.7)
Discussion/activity	41(25.9)	84(40.8)	26(24.5)^**^	101(40.4)	50(22.7)^***^	81(30.3)	70(34.5)	151(32.1)
TV/video	53(33.5)	78(37.7)	33(31.1)	75(30.0)	89(40.5)^*^	97(36.3)	67(33.0)	164(34.9)
Books/newspaper	56(35.4)	96(46.6)	46(43.4)	103(41.2)	95(43.2)	98(36.7)	100(49.3)^**^	198(42.1)
Broadcast/audio	56(35.4)	19(9.2)	13(12.3)^***^	27(10.8)	61(27.7)^***^	58(21.7)	30(14.8)	88(18.7)
Poster/exhibition	8(5.1)	47(22.8)	8(7.55)^***^	30(12.0)	33(15.0)	33(12.4)	30(14.8)	63(13.4)
Internet	49(31.0)	57(27.7)	43(40.6)	77(30.8)	72(32.7)	91(34.1)	58(28.6)	149(31.7)
Counseling	23(14.6)	12(5.8)	13(12.3)^*^	36(14.4)	12(5.45)^**^	17(6.4)	31(15.3)^**^	48(10.2)

*p < 0.05,**p < 0.01 and ***p < 0.001

Significant urban/rural and gender differences were also observed in terms of the sources of and barriers and preferences in accessing SRH information (Table 3).

### Factors associated with SRH knowledge

Table 4 shows factors associated with SRH knowledge scores by type of disability. Among respondents with visual and hearing impairment, urban respondents were more likely to have higher scores than their rural counterparts, and respondents with an education level of senior high school or above significantly scored higher than those with junior high or lower education level. However, only marginally significant association between residential area or education level and SRH knowledge was observed among respondents with physical disability. Respondents aged 20–24 years were more likely to have higher score than their counterparts aged 15–19 years, but this association was observed only in the group with visual impairment and physical disability. Those who were not the single child in the family or whose fathers had received education at senior high level or above were more likely to have higher score than their counterparts in the hearing impaired group, but this association was not observed in the visual and physical impaired groups. Gender, disability onset, living circumstance and mother’s education level were not associated with SRH knowledge.

**Table 4:** Factors associated with SRH knowledge among youth with disabilities, OR(95CI%)

Variables	Visual (n = 158)	Hearing (n = 207)	Physical (n = 108)
*Gender*			
Male	Ref	Ref	Ref
Female	1.73(0.86,3.48)	1.42 (0.77,2.60)	0.88 (0.39,2.02)
*Age group*			
15 ~ 19	Ref	Ref	Ref
20 ~ 24	2.33 (1.19,4.56)^*^	0.83 (0.45,1.52)	2.44(1.08,5.50)^*^
*Residential area*			
Rural	Ref	Ref	Ref
Urban	3.89(1.64,9.23)^**^	3.56 (1.80,7.04)^***^	2.69 (0.93,7.82)
*Single child*			
Yes	Ref	Ref	Ref
No	0.85 (0.40,1.82)	2.19 (1.15,4.17)^*^	1.06(0.44,2.57)
*Education level*			
Junior high or lower	Ref	Ref	Ref
Senior high or above	3.71 (1.33,10.30)^*^	4.96 (2.38,10.37)^***^	1.98 (0.83,4.71)
*Living circumstance*			
With parents	Ref	Ref	Ref
With others	0.79 (0.39,1.60)	1.10 (0.57,2.14)	1.84(0.76,4.44)
*Disability onset*			
From birth	Ref	Ref	Ref
After birth	1.21(0.61,2.42)	1.07 (0.56,2.07)	1.89 (0.80,4.50)
Unknown	0.78 (0.16,3.76)	0.60 (0.25,140)	0.18 (0.05,0.63)^**^
*Father’s education*			
Primary or lower	Ref	Ref	Ref
Junior high	2.11 (0.77,5.77)	1.69 (0.63,4.53)	1.82(0.49,6.79)
Senior high or above	2.44(0.77,7.75)	3.35(1.13,9.92)^*^	1.09 (0.25,4.71)
Unknown	0.44 (0.08,2.36)	3.38(0.89,12.77)	0.27 (0.03,2.51)
*Mother’s education*			
Primary or lower	Ref	Ref	Ref
Junior high	1.76 (0.65,4.80)	1.73 (0.78,3.82)	0.76 (0.25,2.30)
Senior high or above	1.33(0.42,4.23)	1.06 (0.38,3.00)	1.30 (0.33,5.12)
Unknown	1.27 (0.28,5.71)	0.08(0.02,0.28)^***^	1.74(0.21,14.19)
*Parents’ disability*			
None	Ref	Ref	Ref
One or both parents	0.79 (0.35,1.79)	0.88 (0.45,1.72)	0.65 (0.27,1.61)

*p < 0.05; **p < 0.01; ***p < 0.001

## Discussion

This study sought to investigate the knowledge level and access barriers to SRH information among unmarried youth with visual, hearing and physical disabilities in China. The results show that the respondents had limited knowledge and access to SRH information, especially those from rural areas. In general, residential area and education level were significant correlates of knowledge among the respondents. Although the sources of and barriers and preferences in accessing SRH information varied across disability types, the school teachers were the primary and most preferred sources of SRH knowledge.

In this study, the respondents had poor knowledge of SRH, which is consistent with the findings observed in previous studies [31–33]. Among the three categories of knowledge surveyed under this study, the score for STIs/HIV/AIDS was the highest, which might be the result of the implementing HIV/AIDS awareness-raising and prevention education program in the whole country [34].

This study’s findings of the association between types of disability and SRH knowledge were similar to those of the studies conducted in Ethiopia and Ghana [35, 36]. In this study, youth with hearing impairment and physical disabilities had lower level of knowledge than those with visual impairment in all categories of knowledge. Compared with their hearing peers, youth with hearing impairment often face more barriers in accessing SRH knowledge. Due to the barrier in verbal communication, they had less ability in reading comprehension than their counterparts with visual impairment or physical disabilities [33]. Some of them did not even understand such vocabularies as menstruation, nocturnal emission, masturbation, sexual intercourse and marriage [31]. Moreover, youth with hearing impairment are more likely to be isolated from the society than their counterparts with visual impairment or physical disabilities [37]. Possibly due to this reason, respondents with hearing impairment in this study were significantly more likely to get knowledge and information from their peers and report “unaware of the available sources of accurate information” as the key barrier in accessing SRH information than their counterparts with visual impairment or physical disabilities. This study found that that the respondents with hearing impairment who had siblings reported higher level of SRH knowledge than those who were the single child in the family (aOR = 2.94). For respondents with hearing impairment, being a single child in the family is an added disadvantage in accessing SRH knowledge and information. The lower level of knowledge among respondents with physical disabilities than those with visual impairment may be explained by a higher percentage of them having received only junior high or even lower level of education (47.2% vs 15.2%).

Besides school teachers, parents were one of the major sources of information for respondents with disabilities. The finding of this study that the respondents from urban areas had higher level of SRH knowledge than their rural counterparts could be due to their significantly higher level of access to SRH knowledge and information through schools (73.5% vs. 41.4%) and parents (35.2% vs. 26.4%) and less access barrier as demonstrated by a lower percentage of them responding “unaware of the available sources of accurate information” (24.5% vs. 40.9%). The findings from in-depth interviews with the teachers of special education schools and regular schools in urban and rural areas revealed that schools in rural areas were more likely to face challenges than those in urban areas in delivering sexuality education, due to lack of teaching materials and tools, lack of professionally trained teachers, lack of awareness of the importance of sexuality education and lack of support from parents [29]. In rural schools, sexuality-related teaching contents were limited to basic information on physiological anatomy and hygiene, and relationships with the opposite sex, while urban schools provided more relevant information such as puberty change, HIV/AIDS, relationships with the opposite sex, sexual ethics and self-protection [29]. Compared with their urban counterparts, parents in rural areas were usually less educated, more conservative to sexuality education and less likely to provide SRH knowledge and information to their children [30]. Even though almost all parents in urban areas recognized the importance of sexuality education for young people with disabilities and some of them had even communicated with their children about sexuality-related issues, this communication was limited to puberty change, relationships and self-protection with topics such as pregnancy, abortion, contraception and STIs excluded [30]. The fact that the schools and families could not meet the needs of young people for SRH knowledge might explain why the Internet had become the primary preferred source of SRH information for respondents in rural areas. Findings from this study highlight the need to strengthen school and family-based sexuality education.

No gender difference in SRH knowledge was observed by this study, which was also in line with the study conducted in Ethiopia[36]. Age was significantly associated with SRH knowledge; however, this association was not observed among respondents with hearing impairment. The possible explanation for this finding was that among the respondents with hearing impairment, a higher percentage of those aged 20–24 were from rural areas compared with those aged 15–19 (46.6% vs 30.8%).

The relationship between the level of education and SRH knowledge has been documented by many studies [38, 39]. In this study, a strong relationship between the education level and SRH knowledge was also observed among respondents with visual and hearing impairments. The finding that only marginally significant association was observed among respondents with physical disability might be due to small sample size on the one hand, and the high percentage of non-students (over fifty percent) on the other hand. Compared with students, non-students were more likely to be in the age group of 20–24 (72.7% vs 15.1%) and have junior high or lower education (65.5% vs 32.1%). Reports from China and other countries show that not all people with disabilities have access to education [40–42]. According to the data released by the China Disabled Persons' Federation in 2013, only 72.7% of children with disabilities between the ages of 6 and 14 received nine years of compulsory education nationwide [41], compared to 99.7% of children without disabilities [43], a gap of nearly 30% between the two groups. Fortunately, China has made considerable efforts to accelerate the development of special education. Over the past seven years, the number of students enrolled in special education schools had more than doubled from 368,000 in 2013 to 881,000 in 2020 (an increase of 139%), according to statistics released by the Ministry of Education [44]. The Program for Promoting Special Education: Phase II (2017–2020) released by the Ministry of Education and six other state agencies in July 2017 introduced the target of achieving an enrollment rate of over 95% for children with disabilities in compulsory education by 2020 [45]. This makes it possible for young people with disabilities to receive sexuality education at school. Given that school is the most important place to learn knowledge and skills systematically, and that the enrollment rate of children with disabilities in compulsory education is increasing, the promotion of school-based sexuality education should be given priority in China in order to raise the level of SRH among youth with disabilities.

As shown in this study, although differences in the needs and preferences in accessing SRH information were observed across disability types, residential areas and gender, respondents with disabilities had similar SRH information needs and preferred sources. Puberty changes and health care, friendship/love/marriage, sexual harassment/abuse/self-protection and STIs/HIV prevention were the most frequently mentioned topics. School/teachers and the Internet were the most frequently mentioned preferred sources. In the traditional Chinese society, it remains a taboo to talk about sex in public or between different generations [46]. Even if parents realized the importance of sexuality education for their children, only very few communicated with their children about sex-related topics [47]. Not surprisingly, parents were only the third or fourth preferred source of information, as reported by respondents with different characteristics in this study. The most significant difference was observed in ways of obtaining SRH information between urban and rural areas. Classroom teaching/lecturing was the predominant way of acquiring SRH information in urban areas, while books/newspapers and TV/radio were the two leading ways of acquiring SRH information in rural areas possibly due to limited access to school-based sexuality education.

Sexuality education is essential to the health and wellbeing for youth with disabilities. Understanding their needs and access barriers to sexuality education is the first step to address their unmet needs. To the best of our knowledge, this was the first study about the understanding of SRH knowledge, as well as barriers and preferences in accessing sexuality-related information among unmarried youth with different types of disabilities in both urban and rural areas in China. In addition, this study sheds lights on how to address the barriers to sexuality education for youth with different types of disabilities living in the Chinese culture. First, there is an urgent need to raise awareness among relevant government officials, school teachers and educators as well as parents about the importance of providing sexuality education for youth with disabilities, particularly in rural areas. Raising awareness about the SRH needs and rights of youth with disabilities is necessary to overcome stigma, discrimination and misunderstanding. Second, school and family-based sexuality education should be promoted and tailored to the needs of youth with different types of disabilities, with more priority given to school-based sexuality education. Third, policies, systematic supervision and evaluation, disability-friendly teaching and learning materials and relevant curriculum standards should be improved, developed or implemented to support school-based sexuality education. Efforts should be made to support capacity building among teachers and parents. Finally, youth with disabilities must be empowered to seek information proactively.

The study has some limitations. First, given the difficulties in reaching enough respondents with disability, the convenient sampling method was used to recruit participants. Findings in this study may not necessarily represent the overall population of youth with disabilities in China. For example, all of the participants in this study were literate, while only 72.7% of disabled children aged 6–14 received compulsory education nationwide [41], suggesting an even lower level of SRH knowledge and more barriers in accessing sexuality education for the whole population of youth with disabilities. The second limitation was the response bias of self-reporting data. However, efforts were made throughout the process to minimize the bias by ensuring confidentiality and anonymity. Finally, the data of this study was collected several years ago. However, no new relevant data has been made available since then. Although the Chinese government had committed itself to improving education for the reproductive rights of the disabled, there has been little progress in the provision of sexuality education in China during recent years. The challenges of providing sexuality education for children and youth remain in terms of the gaps in the stakeholders’ perspectives and involvement, culture barriers, lack of teaching and learning materials and trained teachers, as well as weak monitoring and evaluation mechanisms[48, 49].

## Conclusion 

Unmarried youth with visual, hearing and physical impairment and disabilities, especially those from rural areas, lacked knowledge about sexual physiology, contraception and STIs/HIV/AIDS, and had limited access to SRH information through schools and families. Limited sources of information and the embarrassment in seeking information under the influence of the Confucian culture deeply-rooted in the Chinese society were the two most common barriers they faced in accessing SRH information. Despite limited access to SRH information through school, the school teachers were still the most preferred source of SRH knowledge. This study highlights the barriers faced by youth with disabilities in accessing SRH knowledge and information in the Chinese culture and sheds some light on the potential strategies to reduce the barriers. To address their unmet needs for SRH information, there is an urgent need to raise awareness among relevant government officials, school teachers, educators and parents about the importance of providing sexuality education for youth with disabilities. Furthermore, efforts should be made to support the delivery of sexuality education by schools and families and empower young people with disabilities to seek information proactively.

## Data Availability

The datasets analyzed during the current study are available from the corresponding author on reasonable request.
